# Supplementary material for: Collaborative intelligence in AI: Evaluating the performance of a council of AIs on the USMLE
Source: PLOS Digit Health. 2025 Oct 9;4(10):e0000787. doi: 10.1371/journal.pdig.0000787 (PMC12510544; doi:10.1371/journal.pdig.0000787)
Supplement: S2 File — (ZIP) [file pdig.0000787.s002.zip › S2 Data Raw data files containing unprocessed question inputs and LLM outputs including transcripts of deliberations/STEP3_Question-56-1_Type-MC-NJ.docx]

**Facilitator** **[Round 1]:** A 45-year-old man comes to the community health center because of a 1-week history of fever, chills, sweating, mild shortness of breath, and nonproductive cough. Medical history is remarkable for AIDS and pneumonia 3 years ago that required hospitalization. Today, he says that he lost his job 6 months ago and cannot afford to buy his antiretroviral medications. Vital signs are temperature 38.6°C (101.5°F), pulse 82/min, respirations 20/min, and blood pressure 116/72 mmHg. Pulse oximetry on room air shows an oxygen saturation of 97%. The health center is very busy with several patients waiting to be evaluated. Which of the following is the most appropriate next step? (A) Arrange a direct admission to the hospital for this patient (B) Continue with obtaining a thorough history and examining this patient (C) Place masks on the patient and yourself and then continue to evaluate him (D) Send this patient for chest x-rays, and while they are being obtained examine the next patient

**AI_E:** (1) RESTATING THE QUESTION: The question asks about the most appropriate next step in managing a 45-year-old male patient who presents with symptoms indicating a potential respiratory infection. The patient has a medical history significant for AIDS and pneumonia, has been unable to afford antiretroviral medications due to recent job loss, and currently has fever and respiratory symptoms. The choices provided indicate different approaches including immediate hospital admission, thorough examination, infection control measures, or diagnostic investigation alongside management of other waiting patients. (2) PLANNING: Assumptions here may include the possibility of a severe or communicable respiratory infection, importance of in-depth history and physical examination, potential risk to the healthcare provider, or efficient use of time. Alternate assumptions could include the possibility of mild self-limiting symptoms, or the risk of overlooking other important conditions due to focus on the most obvious symptoms. The priority here, given the patient's history and symptoms, could arguably be (1) resolving uncertainty about his diagnosis and (2) evaluating the severity of his condition to consider immediate hospitalization. (3) CLINICAL REASONING: His history of AIDS, unchecked due to loss of antiretroviral treatment, increases his susceptibility to infections, including pneumonia. His symptoms could suggest a respiratory infection, which may be worsened by his immuno-compromised state. Additionally, assessment of how well his body is dealing with this potential infection is essential, given the elevated temperature. Physical examination and medical history are fundamental diagnostic tools in determining the severity and exact nature of his symptoms. Although there's a crowded waiting room, given the potential severity of his condition, it is important to focus on him rather than risk his health deteriorating. (4) CONCLUDING: Considering his medical history, the potential for a severe infection, and his current symptoms, the most appropriate next action is a thorough assessment of the patient. This will not only guide diagnosis but also allow for judgement on necessary interventions. (5) RESPONSE: Therefore, the best course of action is to (B) Continue with obtaining a thorough history and examining this patient.

**AI_A:** RESTATING THE QUESTION: We are considering the best course of action for a 45-year-old man with AIDS presenting with symptoms that could suggest a serious infection. His financial struggles have interrupted his antiretroviral treatment, exposing him to increased risk of opportunistic infections. We know his vital signs are stable, but the clinic is busy and we must consider appropriate measures for everyone's safety. PLANNING: Assumptions that could be made include: he may be suffering from an opportunistic infection due to his immunocompromised status, his symptoms seem non-life-threatening for now, but could progress, he may be infectious to others in the care setting due to his symptoms, the facility resources may be limited and need to be managed appropriately. An alternate assumption considering his comprehensive clinical picture leans towards a potentially serious opportunistic infection due to his untreated AIDS condition, among other vulnerable patients. CLINICAL REASONING: Firstly, although his vital signs are somewhat stable now, with AIDS and no current antiretroviral therapy, he's at risk of serious or even life-threatening opportunistic infections such as Pneumocystis pneumonia. Therefore, his condition must not be considered as non-urgent. Secondly, his symptoms like fever, cough may be contagious, and close contact without proper precautions could increase the transmission risk in a healthcare setting, even though the exact pathogen is not yet identified. Lastly, considering the other patients waiting, the importance of maintaining the workflow of the clinic should not be overlooked. CONCLUDING: Based on the considerations given above, it would be judicious to prioritize patient safety, infection control measures, while also facilitating his in-depth diagnosis and management. Within these perspectives, him potentially having an infectious condition and the crowded health center scenario propound the need for infection control without compromising healthcare delivery to others. RESPONSE: Therefore, the most appropriate next step would be to (C) Place masks on the patient and yourself and then continue to evaluate him - to inhibit potential pathogen transmission and continue with the assessment towards an accurate diagnosis and treatment. While this approach concurrently ensures that clinic workflow is not hampered and cares for other patients awaiting evaluation is not compromised.

**AI_C:** RESTATING THE QUESTION: The given scenario discusses a 45-year-old man, with a notable medical history of AIDS and a prior pneumonia that required hospital admission, now presenting with symptoms suggestive of a respiratory infection. Inadequate access to antiretroviral drugs due to financial constraints is highlighted. The physician is asked to identify the most appropriate next step from four given options, amid the challenges of a busy health center. PLANNING: The scenario presents the typical clinical picture of a patient potentially experiencing a bout of opportunistic infection - a common occurrence in AIDS patients with suboptimal antiretroviral therapy. The first assumption might be that this patient requires immediate hospital admission given his high-risk history. The alternate assumption could be that with suitable precautions, the patient can be assessed in the outpatient setting itself, balancing efficacy of care with optimal resource utilization. The critical factor influencing this decision includes not only the patient's current clinical status but also the potential risk of transmission to others in the health center. CLINICAL REASONING: Assessments must consider the patient's symptoms, the severity of the underlying disease (AIDS), and the substantial risk of life-threatening, opportunistic infections due to his inability to afford antiretroviral therapy. His stable vitals signs and pulse oximetry result, albeit symptomatic, suggest that he is not in immediate respiratory distress, which opens the possibility of out-patient management. However, we must also consider the busy healthcare setting and the infectious nature of his potential condition, leading to an imperative to practice infection control and minimize the risk of transmission. CONCLUDING: Balancing risk management, infection control, and optimal resource utilization, immediate and comprehensive evaluation including history, clinical examination, and appropriate investigations are warranted. This should be carried out with appropriate precautions to prevent the potential spread of infection. RESPONSE: Thus, the most appropriate next step would be to (C) Place masks on the patient and yourself and then continue to evaluate him, taking into account both the patient's potential infectious status and the necessity of ongoing medical assessment for a comprehensive clinical picture.

**AI_B:** RESTATING THE QUESTION: The question presents a 45-year-old man who is suffering from fever, chills, sweating, nonproductive cough and slight shortness of breath for the past week. He has a medical history of AIDS (Acquired Immune Deficiency Syndrome, a condition that compromises the immune system) and pneumonia that required hospitalization 3 years ago. He also reports losing his job and not being able to afford antiretroviral drugs (used to manage HIV infection and prevent AIDS progression). His vitals indicate a fever, but otherwise normal. Despite an oxygen saturation of 97%, the health center is quite crowded. We are asked to decide the most appropriate next step, with options ranging from admitting this patient directly to the hospital, proceeding with obtaining a thorough history and examining this patient, taking precautions against airborne infections or sending him for chest x-rays. PLANNING: The assumptions can be that he may be suffering from the same pneumonia again due to a decrease in immunity from not taking antiretroviral drugs for 6 months or his symptoms might be due to a new HIV-related opportunistic infection. Another assumption could be that the patient's condition might worsen without immediate intervention given his AIDS status. It would also be worth considering that his symptoms could merely be due to a common cold or flu and not necessarily indicative of something more serious. To select the most appropriate action, we must prioritize the assumption that takes into account the patient's health status, clinic status as well as infection control. CLINICAL REASONING: The above-mentioned symptoms might be the result of a resurgence of a pneumonia or a manifestation of another opportunistic infection common in AIDS patients such as Pneumocystis jiroveci pneumonia, Tuberculosis, etc. These infections are quite severe in immunocompromised individuals and can rapidly deteriorate their health if not addressed immediately. Besides, this patient has a history of pneumonia requiring hospitalization which further supports this assumption. However, this must be balanced against the need for timely evaluation of other patients and the possible risk of transmitting infection within the health center. CONCLUDING: Considering the patient's medical history, compromised immune status, and the severity of his symptoms, the initial implication seems to lean towards infection control and further evaluation of the patient. This does not rule out potential future needs for chest x-rays or possible hospital admission but indicates the prioritization of immediate actions based on the presumed risk of infection and the patient's current health status. RESPONSE: Therefore, the most appropriate next step would be option (C) place masks on the patient and yourself and then continue to evaluate him. This combines both precautions against potential infection transmission, given his history of pneumonia and AIDS, and further examination and diagnosis. Further investigations can be planned based on findings from the history and examination. It will help to identify if the patient has a recurrence of a previously diagnosed infection or a new one due to the compromised immune system. Once additional data is collected, decisions about necessary interventions, which could include chest x-rays or hospitalization, can be made on a more informed basis.

**AI_D:** RESTATING THE QUESTION: To recap, we have a 45-year-old man who presented to a health center with a week-long history of fever, chills, sweats, breath discomfort, and a dry cough. Notably, he has a history of AIDS and previous hospitalization due to pneumonia. He also reported that he lost his employment and has been unable to maintain his antiretroviral treatment regimen because of financial constraints. The patient's body temperature is elevated, but his other vital signs are within normal limits. With the center busier than usual, the question is what is the most appropriate next step from the following options: hospital admission, further history and exam, wearing masks and continued evaluation, or sending him for a chest x-ray before examining the next patient? PLANNING: The implicit assumption is that the patient's symptoms are relating to his HIV infection and his inability to access his antiretroviral therapy. However, looking from another assumption, he could potentially be suffering from another pathogen or non-infectious cause like malignancy. Patients with uncontrolled HIV are at risk for a variety of opportunistic infections and malignancies because of their immunocompromised state. Hence, a comprehensive evaluation is important in this patient for appropriate management. Therefore, the best course of action could be to maximize clinical information through a thorough history and physical exam. CLINICAL REASONING: Considering the patient's history of AIDS and non-management, he is at an increased risk for opportunistic infections, which can present similarly to his current symptoms. Given his concurrent fever and respiratory symptoms, an infective cause like pneumonia is one possibility. However, with his history of AIDS, we cannot overlook the potential for more serious, opportunistic infections such as Pneumocystis pneumonia, Tuberculosis or even malignancies that are more prevalent in this population, such as Kaposi's sarcoma or Non-Hodgkin lymphoma. To make a judicious determination, a more extensive history, and a thorough physical examination is traditionally the first step in evaluating any patient. CONCLUDING: Arriving at a working diagnosis via clinical history and examination is the cornerstone of managing a patient effectively. Hospital admission or sending him for an X-ray before proper evaluation would not be routinely done unless the patient's vital signs were unstable. The use of masks would be appropriate in a patient with a highly contagious disease. However, there is no information suggesting that his current symptomatology could be a result of such an infection. RESPONSE: So, the most appropriate next step in managing this patient would be option B: continue with obtaining a thorough history and examining this patient. This will allow us to apprise the severity and potential cause of his fever and respiratory symptoms, which are crucial for planning further investigations and management.

**Facilitator** **[Round 2]:** Summary: AI_A believes that the patient's symptoms, combined with his untreated AIDS condition, suggest a potentially serious opportunistic infection. They recommend prioritizing patient safety and infection control measures, while also facilitating his in-depth diagnosis and management. They suggest placing masks on the patient and the healthcare provider to inhibit potential pathogen transmission and continue with the assessment towards an accurate diagnosis and treatment. AI_B also suggests placing masks on the patient and the healthcare provider, but emphasizes the need for further examination and diagnosis. They believe that the patient's symptoms might be due to a resurgence of pneumonia or a new HIV-related opportunistic infection, and that immediate intervention is necessary given his AIDS status. AI_C agrees with the need for masks and further evaluation, but also highlights the importance of balancing risk management, infection control, and optimal resource utilization. They believe that immediate and comprehensive evaluation, including history, clinical examination, and appropriate investigations, are warranted. AI_D, however, believes that the most appropriate next step is to continue with obtaining a thorough history and examining the patient. They argue that hospital admission or sending him for an X-ray before proper evaluation would not be routinely done unless the patient's vital signs were unstable. AI_E also suggests continuing with obtaining a thorough history and examining the patient, arguing that resolving uncertainty about his diagnosis and evaluating the severity of his condition should be the priority. Key Differences: The key differences in the council members' opinions lie in their prioritization of next steps. AI_A, AI_B, and AI_C prioritize infection control measures and further evaluation, while AI_D and AI_E prioritize obtaining a thorough history and examining the patient. Question for Council Members: What is the basis for your prioritization of either infection control measures and further evaluation (AI_A, AI_B, AI_C) or obtaining a thorough history and examining the patient (AI_D, AI_E)? How do you weigh the potential risks and benefits of these different approaches in this specific case?. Based on your preceding response, how would you answer the original question: [A 45-year-old man comes to the community health center because of a 1-week history of fever, chills, sweating, mild shortness of breath, and nonproductive cough. Medical history is remarkable for AIDS and pneumonia 3 years ago that required hospitalization. Today, he says that he lost his job 6 months ago and cannot afford to buy his antiretroviral medications. Vital signs are temperature 38.6°C (101.5°F), pulse 82/min, respirations 20/min, and blood pressure 116/72 mmHg. Pulse oximetry on room air shows an oxygen saturation of 97%. The health center is very busy with several patients waiting to be evaluated. Which of the following is the most appropriate next step? (A) Arrange a direct admission to the hospital for this patient (B) Continue with obtaining a thorough history and examining this patient (C) Place masks on the patient and yourself and then continue to evaluate him (D) Send this patient for chest x-rays, and while they are being obtained examine the next patient]

**AI_A:** RESTATING THE QUESTION: We’re deciding on the immediate approach for a 45-year-old man with a high fever, cough, shortness of breath, and a history of AIDS and pneumonia. He’s not currently on antiretroviral medication due to financial issues. We’re selecting from four options: admitting him directly to the hospital, performing a more detailed history and physical exam, taking infection control measures and then proceeding with evaluation, or ordering a chest X-ray while seeing other patients in the meantime. PLANNING: The assumptions here are that (1) the man's symptoms are severe enough to require hospitalization, (2) more information will improve the specificity of our diagnosis, (3) the prevention of potential infection transmission commands prioritization, and (4) the severity of the man's symptoms, his AIDS status, and the unavailability of antiretroviral therapy necessitate specific diagnostic imaging before moving forward. One alternate assumption could be that his symptoms, history, and lack of current treatment might indicate an infectious process, and potential risk of transmission to others, necessitating an immediate infection control approach. CLINICAL REASONING: With a clinical history of AIDS, pneumonia, and presenting symptoms such as fever, chills, shortness of breath, and cough, this individual possibly has an opportunistic infection. The non-availability of antiretroviral treatment likely adds to his immunocompromised state, making him more susceptible to infections, including respiratory ones. Generally, these can be transmitted via droplets or aerosols, posing a risk to healthcare staff and other patients in close proximity. Therefore, preventing potential pathogen transmission while evaluating the patient extensively for an accurate diagnosis would be a relevant clinical approach. CONCLUDING: Considering the potential severity of his undisclosed condition, the risk to healthcare staff and other patients, and the urgent need for a detailed assessment to plan further management, placing masks on the patient and ourselves before continuing the evaluation would be a reasonable option. This step would ensure infection control, while also allowing for a clinically relevant thorough evaluation. RESPONSE: Given the reasons supported above, my response of choice would be "Place masks on the patient and yourself and then continue to evaluate him" (Option C). This response prioritizes both infection control and the comprehensive assessment necessary for the proper diagnosis and treatment of this patient.

**AI_C:** The question at hand pertains to the management of an immunocompromised patient presenting with respiratory symptoms which could indicate an opportunistic infection. In this clinical scenario, two primary factors must be considered upon which the entire scenario pivots: (1) the patient's current clinical presentation and health status; (2) the epidemiological perspective, specifically related to infectious disease control in a health center given the patient's untreated HIV status. Keeping in line with the established pattern, starting with restating the question: The patient's untreated AIDS status heightens his susceptibility to unusual infections and malignancies, reflected in his past history of pneumonia and the recent onset of respiratory symptoms suggestive of a possible pulmonary infection. Moving onto the planning stage: A key assumption in this scenario relates to the potential contagious nature of the patient's symptoms. The severity of the patient's condition and the need for immediate intervention or further diagnostic evaluation are also at play. These assumptions are hinged on balancing the risk of potential pathogen transmission to others, managing the patient’s immediate health needs, respecting the workload and patient safety protocols in a busy health center. Next, clinical reasoning: An untreated AIDS patient with a fever, chills, sweating, mild shortness of breath, and a nonproductive cough is likely suffering from an opportunistic infection, which may potentially be infectious to others, especially in a closed healthcare setting. Given the general public health implications, measures to prevent pathogen transmission should be put in place whilst ensuring the patient is being properly assessed and treated. Detailed history taking and physical examination are necessary to gauge the severity of his condition and guide further investigations and management. This brings us to the conclusion stage: Prioritizing the safety of healthcare staff and other patients, along with meeting the healthcare need of this patient, we converge upon a path that ensures pathogen transmission prevention, and quality clinical care for the patient. Hence, in response to the specific question, the most appropriate next step would be to "Place masks on the patient and yourself and then continue to evaluate him" (Option C). The use of masks will reduce the chance of pathogen transmission, and continuing evaluation will provide the necessary clinical particulars to guide diagnosis and management. While arranging a chest X-ray is beneficial for diagnostic purposes, the basic clinical evaluation should not be deferred, and direct admission should be decided based on the severity of his condition, hence, emphasizing the significance of a thorough clinical evaluation.

**AI_D:** As 'AI_D', my view is based on the premise of standard clinical practice, in which thorough history taking and physical examination form the cornerstone of medical evaluation and diagnosis (Restating the Question). My suggestion to proceed with these steps first stems from specific assumptions: while the patient's symptoms and remarkable medical history indeed raise alarm for potential opportunistic infection due to untreated AIDS, the clinical presentation at this moment does not seemingly indicate immediate life-threatening emergency due to its subacute nature and stable vital signs. Therefore, the urgency of immediate protective interventions, such as sending the patient for chest X-ray or arranging hospital admission, seems less (Planning). In questioning these assumptions, one could argue that the patient’s immunocompromised status demands immediate protective measures, such as masking or expediting diagnosis via radiology (Generating Alternates). However, given the limited resources of the community health center and the multiple waiting patients, it would be more pragmatic to prioritise a detailed clinical evaluation to establish a better understanding of the patient’s condition and his potential risk to his environment (Evaluating Alternates). Now, applying clinical reasoning to our foreground case, we know that HIV/AIDS patients can develop a variety of opportunistic infections, and early recognition of these conditions can improve clinical outcome. Particularly if they exhibit flu-like symptoms, this may be indicative of diseases like Tuberculosis (TB), which can be highly communicable. However, while this patient does have a potential risk of such infections, his current stable vitals, normal oxygen saturation, and subacute symptomatology do not immediately suggest severe pulmonary contagion like active TB (Clinical Reasoning). In conclusion, my suggested approach aligns with the patient-centered care model that emphasizes a comprehensive understanding of the patient's condition and personal context before initiating any intervention. Prioritizing this process above routine infection control measures in an otherwise stable patient also allows for a careful allocation of the center's busy resources while minimizing potential panic or stigmatization (Concluding). As such, my final response would be to choose option (B): Continue with obtaining a thorough history and examining this patient. This approach allows us to optimize the patient’s own safety and wellbeing while mitigating potential communal risk and ensures a targeted and individualized use of the health center's limited resources (Response).

**AI_E:** To address the question, restating it in simpler terms, we're to determine what is the most appropriate next step when a 45-year-old man who is untreated for AIDS and has a medical history of pneumonia presents with symptoms such as fever, chills, sweating, mild shortness of breath, and nonproductive cough. The options include hospital admission, getting more history and examining him, employing infection-preventive measures and continuing with his evaluation, or sending the patient for chest X-rays. The patient's symptoms, alongside his medical history and untreated HIV status, could be indicative of several possible clinical scenarios. For planning, these may include a new opportunistic infection, a resurgence of previous pneumonia, or an unrelated pulmonary pathology. Each of these possibilities is contingent on different sets of assumptions about his pathology, requiring us to evaluate each one. The clinical reasoning unfolds like this: If we hypothesize an opportunistic infection due to uncontrolled HIV, this may predispose the patient to specific respiratory pathogens that we wouldn't typically expect in an immunocompetent individual. Nevertheless, the patient's vital signs seem relatively stable, which could make urgent hospitalization possibly unnecessary at this stage. This stability doesn't rule out the need for urgent interventions, as individuals with HIV can present with more subtle signs despite severe infections. Infection control measures might be advisable, as the patient might harbor airborne pathogens such as Tuberculosis, common in HIV patients. This assumption would suggest prioritizing option C—placing masks on the patient and the healthcare provider and continuing to evaluate him. On the other hand, supposing a comeback of his prior pneumonia, or some other non-HIV related lung pathology, this would prompt a thorough evaluation of his history and a comprehensive physical examination, lending weight towards choosing option B–continue obtaining a thorough history and examining the patient. On balance, given the risk of potential infection transmission and the patient's immunocompromised status, the combined strategy of infection control measures and performing an appropriate examination and history-taking seems most prudent. So, to wrap up, while there are several possible clinical indications in this patient due to his untreated AIDS and pneumonia history, given the presented scenario and his relatively stable vital signs, the most appropriate next step seems to be placing masks on the patient and yourself and then continuing his evaluation (C). It incorporates both infection control measures and the necessity of a detailed history-taking and examination, ensuring the safety of healthcare providers and other patients while prioritizing patient care.

**AI_B:** Restating the question: The patient is a 45-year-old male with a history of AIDS and pneumonia, presenting with a week-long history of fever, chills, sweating, shortness of breath, and cough. His AIDS treatment has been interrupted due to financial constraints. Although he has a fever, his other vital signs, including oxygen saturation, are within normal limits. The health center is currently busy with several waiting patients. The query is about the most appropriate next step—whether that's hospital admission, further in-depth history and physical examination of the patient, implementation of infection control measures, or obtaining a chest X-ray while assessing the next patient. Planning: The patient's immunocompromised status due to untreated AIDS and his presenting symptoms could suggest a potential opportunistic infection. The ambiguity lies in the balance between quick and efficient patient flow, thorough patient care, infection control, and optimal resource allocation. Prioritizing clinical reasoning can result in different scenarios: admitting the patient to the hospital; continuing with a comprehensive history and physical examination; implementing infection control measures by donning masks and continuing the evaluation; or sending the patient for a chest X-ray, assuming the presence of a respiratory infection, and seeing the next patient during the process. Clinical reasoning: Given that his symptoms have been present for a week, an infection with an opportunistic pathogen is likely the cause. However, the patient's stable vital signs indicate that any infection might not be severe yet. We cannot rule out an infiltration of the pneumonia that hospitalized him three years ago or a new HIV-related opportunistic infection. Immediate intervention may be necessary due to his untreated AIDS, but hospital admission would usually require a more urgent presentation. Considering the volume of waiting patients and the potential risk of communicable infection, efficient patient flow is essential. Promptly determining the cause of the patient's symptoms is also important to provide necessary treatment and prevent any deterioration. Therefore, assessing this patient thoroughly can prioritize his immediate health needs while simultaneously controlling infection transmission and maintaining an efficient workflow at the health center. Concluding: In this case, therefore, the balance between infection control, patient flow, and thoroughness of evaluation should be navigated by taking immediate infection control measures in parallel with continuing a comprehensive evaluation of the patient. This approach ensures adequate patient care without compromising safety or efficiency. Response: Based on the clinical reasoning, I propose opting for option (C): Place masks on the patient and yourself and then continue to evaluate him. Infection control measures are crucial given the patient's immunocompromised status and presenting symptoms, and further evaluation allows for a better understanding of his current health situation. This approach addresses the potential risk of infection transmission, the patient's immediate health needs, and the vaguity surrounding his condition without disrupting the patient flow in the busy health center.

**Facilitator** **[Round 3]:** Summary: AI_A, AI_B, AI_C, and AI_E all agree on the approach of placing masks on the patient and themselves before continuing the evaluation (Option C). They all emphasize the importance of infection control due to the patient's immunocompromised status and the potential risk of transmission to others. They also highlight the need for a comprehensive assessment to guide further management. AI_D, however, suggests continuing with obtaining a thorough history and examining the patient (Option B). AI_D argues that the patient's stable vital signs and subacute symptomatology do not immediately suggest a severe pulmonary contagion. Therefore, a detailed clinical evaluation should be prioritized to establish a better understanding of the patient’s condition and his potential risk to his environment. Key Differences: The key difference lies in the prioritization of immediate infection control measures versus a thorough clinical evaluation. AI_A, AI_B, AI_C, and AI_E prioritize infection control due to the potential risk of transmission, while AI_D prioritizes a detailed clinical evaluation based on the patient's current stable condition. Question to Clarify Disagreements: Given the patient's immunocompromised status and potential risk of transmission, how do we balance the need for immediate infection control measures with the need for a thorough clinical evaluation? Can these two approaches be effectively combined without compromising the quality of patient care or the safety of healthcare providers and other patients?. Based on your preceding response, how would you answer the original question: [A 45-year-old man comes to the community health center because of a 1-week history of fever, chills, sweating, mild shortness of breath, and nonproductive cough. Medical history is remarkable for AIDS and pneumonia 3 years ago that required hospitalization. Today, he says that he lost his job 6 months ago and cannot afford to buy his antiretroviral medications. Vital signs are temperature 38.6°C (101.5°F), pulse 82/min, respirations 20/min, and blood pressure 116/72 mmHg. Pulse oximetry on room air shows an oxygen saturation of 97%. The health center is very busy with several patients waiting to be evaluated. Which of the following is the most appropriate next step? (A) Arrange a direct admission to the hospital for this patient (B) Continue with obtaining a thorough history and examining this patient (C) Place masks on the patient and yourself and then continue to evaluate him (D) Send this patient for chest x-rays, and while they are being obtained examine the next patient]

**AI_D:** Broadly speaking, the question is asking what should be the most appropriate next step in the management of a 45-year-old immunocompromised man (due to AIDS), who is currently not on antiretroviral therapy, visiting a busy health center due to signs and symptoms consistent with a potential infectious process. The options generally revolve around providing prompt medical attention, ensuring infection control measures, or organizing further diagnostic investigations. If we assume that the major concern is prevention of immediate transmission of infectious agents to the healthcare team or the surrounding patients, then the practice of placing masks on the patient and yourself before continuing the evaluation (Option C) becomes crucial. Alternatively, if the assumption is that a comprehensive clinical evaluation may unveil more information about the patient's current state and guide subsequent investigation and management, we may prioritize taking a detailed history and examining the patient (Option B). When it comes to reasoning clinically, it's important to balance the need for infection control with thorough clinical evaluation especially considering an immunocompromised patient presenting with fever and respiratory symptoms. In this case, the patient's stable vital signs indicate that he does not currently require immediate life-saving interventions. Detailed evaluation will provide valuable information for diagnosing the patient's current condition, informing about his potential risk to others, and tailoring the subsequent management strategy, which may include further investigations, initiation of treatment or hospitalization. Following this step-by-step analysis, it can be concluded that it is appropriate to combine the prioritization of infection prevention measures with the necessity of thorough clinical assessment. Therefore, my response would be to place masks on the patient and yourself, and then continue evaluating the patient (Option C). By doing so we are not only ensuring the safety of others in the health center, but also progressing towards obtaining a detailed clinical picture of the patient's condition which will guide further decisions. This balances potential transmission risks whilst allowing us to continue establishing a diagnosis and implementing appropriate care for the patient.

**AI_E:** Restating the question in my own words, we are asked to decide the best initial approach in assessing a 45-year-old HIV positive man who presents with symptoms suggestive of a respiratory tract infection. As he has been unable to afford his antiretroviral medications, his immune system is likely highly compromised. The choices explore different patient management strategies including hospital admission, detailed history and examination, infection control measures, and diagnostic imaging. The patient's presentation indicates a potential infectious process. Given his HIV status, he is immunocompromised and may present with uncommon infections. Thus, when generating assumption, we must consider that his condition might be contagious and pose a risk to others at the health center. An alternative assumption is that given his history and symptoms, his illness might not be acutely exacerbating or immediately contagious. Given these assumptions, conducting a detailed patient history and examination alone, as stated in option B, may not sufficiently address the potential risks. On the other hand, placing masks on both the patient and healthcare provider before continuing the evaluation, as suggested in option C, provides an added layer of infection control while allowing the provision of care to continue. To conclude, as healthcare providers, we also have an obligation to minimize potential healthcare-associated transmissions. While a thorough history and examination are necessary to guide further management, immediate measures to protect the health center's staff and patients should also be taken. This approach fosters a safer environment for everyone while the patient receives the required care. Therefore, it would be appropriate to select option C: "Place masks on the patient and yourself and then continue to evaluate him." This approach takes into account both the patient's need for care and the requirement for preventing potential transmission of infectious agents in the health center. After implementing proper infection control measures, the healthcare provider can then execute a comprehensive evaluation of the patient's condition.

**AI_B:** RESTATING THE QUESTION: The patient, a 45-year-old man, with a known history of AIDS and pneumonia, presents with a week's history of fever, chills, sweats, mild shortness of breath, and nonproductive cough. These symptoms indicate a possible infectious disease. The decision needs to be made on the most appropriate next step in his management, considering his past medical history, recent loss of antiretroviral therapy, current signs, and symptoms, and the healthcare center's busyness. PLANNING: Several assumptions could be made from the given scenario. One could assume that: a. The patient has contracted a new pulmonary infection due to his immunocompromised status. b. The patient's symptoms are not severe enough to require immediate hospitalization. c. The patient might pose an infection risk for other patients and healthcare providers at the clinic. d. The immediate priority should be to prevent the possible spread of infection versus understanding the root cause of the patient's symptoms. CLINICAL REASONING: The patient's history of AIDS and cessation of antiretroviral treatment significantly predispose him to various opportunistic infections. This susceptibility, combined with the patient's current symptoms, strongly suggest the possibility of a new infection, possibly pulmonary in nature. Considering his past history of pneumonia, it is essential to evaluate his current condition thoroughly. However, given the busy nature of the health center and the patient's possible infectious state, it becomes pertinent to prioritize infection control measures, for the patient's sake and for all other individuals in the vicinity. CONCLUDING: Balancing the need for comprehensive clinical evaluation and immediate infection control measures, the most appropriate next step would be to equip the patient and healthcare providers with masks and then continue evaluating him. RESPONSE: Option (C) - Place masks on the patient and yourself and then continue to evaluate him. This approach ensures both the continuity of clinical assessment, addressing the patient's healthcare needs, and infection control, fulfilling the obligation of do-no-harm to others in the healthcare setting.

**AI_A:** (1) RESTATING THE QUESTION: The inquiry revolves around a 45-year-old man with a history of acquired immunodeficiency syndrome (AIDS) and pneumonia who is displaying symptoms such as fever, chills, sweating, shortness of breath, and a non-productive cough. Currently, he is unable to afford his antiretroviral medications. He has visited a health center which is presently busy with other patients. The question asks which would be the most suitable next step to proceed with in this scenario from a given set of options. (2) PLANNING: Given the available options, the underlying assumptions vary. The decision can be influenced by various clinical considerations like managing potential contagion, the severity of the patient's current symptoms, the patient's history, and the potential impact of the chosen approach on the patient's overall management and other patients at the health center. The assumptions that can be made are: A) The patient's condition mandates an immediate hospitalization. B) The patient requires a more comprehensive history and examination before deciding on the next step. C) Preventing the potential transmission of an infectious pathogen should be the immediate priority. D) Obtaining chest x-rays and managing the patient load should be prioritized. After identifying these assumptions, I find that prioritizing infection control and comprehensive assessment (option C) should be the initial clinical management strategy. (3) CLINICAL REASONING: Based on his symptoms and medical history, the patient is likely suffering from an infectious disease, which can potentially be transmitted to others. This is especially relevant given his immunocompromised state due to AIDS. Therefore, slowing the potential spread of infection - through the implementation of infection control measures such as mask wearing - is important. Simultaneously, obtaining a comprehensive history and performing a thorough physical examination is crucial in guiding the subsequent management steps, including whether hospitalization, chest x-rays or any other interventions are warranted. (4) CONCLUDING: In essence, since the patient's vital signs are stable and he is hypoxemic, taking the necessary precautions to prevent any potential spread of an infectious pathogen while simultaneously gaining a better insight into his condition appears to be the most balanced and clinically justified approach. (5) RESPONSE: Therefore, I would choose option C: Place masks on the patient and yourself and then continue to evaluate him. This approach enables us to assess the severity and nature of the patient's condition, while also preventing potential transmission of infectious pathogens due to his immunocompromised state.

**AI_C:** RESTATING THE QUESTION: We have a 45-year-old male patient with a history of AIDS and non-compliant antiretroviral therapy (ART) since the last 6 months due to financial issues. He has been experiencing fever, chills, sweating, mild shortness of breath, and nonproductive cough for a week. The patient’s vital signs indicate that he is febrile but otherwise stable. In this busy healthcare setting, our task is to decide the most appropriate next step in managing this patient. PLANNING: The four options can be categorized into immediate hospitalization (A), comprehensive clinical evaluation (B), infection control (C), or parallel processes – proceeding with a chest x-ray while seeing the next patient (D). Considering the patient’s symptoms, stable vital signs, history of AIDS and pneumonia, and his non-compliance with antiretroviral therapy, we must examine the potentials for a re-emergence of an opportunistic infection and possibilities of transmission to other patients and healthcare providers. CLINICAL REASONING: The fever and respiratory symptoms could indicate the re-emergence of an opportunistic infection due to the patient not taking his antiretroviral medications. As such, the re-emergence of pneumonia – whether bacterial, fungal, or viral – is a valid assumption, especially considering the patient's history. Hospitals are usually reserved for severe or drastically worsening conditions – while this patient is experiencing discomfort, its severity and its acute or subacute presentation do not warrant immediate hospitalization as per his vital signs. The patient's history and symptomatology do, however, warrant a thorough clinical examination and preliminary investigations. CONCLUDING: Consequently, while diagnosing the exact nature of his illness is not immediately possible without further diagnostic steps, prioritizing infection control practices as a precautionary measure seems to be an optimal approach. This approach ensures the safety of the staff and other patients while we prepare to proceed with a further clinical evaluation and diagnostics. RESPONSE: Given this frame of thought, the most appropriate next step would be to place masks on the patient and yourself and then continue to evaluate him (Option C). This approach provides a balanced solution, taking into account the immediate need for infection control measures, due to the immunocompromised status of the patient, while not skimping on a thorough assessment to formulate a comprehensive management plan.

**Facilitator** The consensus among the AI responses is that the most appropriate next step in managing the 45-year-old man with a history of AIDS and pneumonia, who is presenting with symptoms suggestive of a potential infectious disease, is to prioritize infection control measures while continuing with his evaluation. This approach, represented by option C, involves placing masks on both the patient and the healthcare provider before proceeding with the assessment. This decision is based on several considerations, including the patient's immunocompromised state due to his AIDS condition and non-compliance with antiretroviral therapy, his current symptoms indicative of a possible infectious process, and the busy nature of the health center. The consensus acknowledges the importance of a comprehensive clinical evaluation to guide subsequent management steps, but emphasizes the immediate need to minimize potential transmission risks to other patients and healthcare providers in the setting. Therefore, the consensus response to the prompt is to choose option C: "Place masks on the patient and yourself and then continue to evaluate him."
